# Anti-Hyperglycemic Effects of Thai Herbal Medicines

**DOI:** 10.3390/plants13202862

**Published:** 2024-10-13

**Authors:** Athit Bunyakitcharoen, Weerakit Taychaworaditsakul, Seewaboon Sireeratawong, Sunee Chansakaow

**Affiliations:** 1 Department of Pharmaceutical Sciences, Faculty of Pharmacy, Chiang Mai University, Chiang Mai 50200, Thailand; athit_b@cmu.ac.th; 2 Department of Biochemistry, Faculty of Medicine, Chiang Mai University, Chiang Mai 50200, Thailand; weerakit.tay@cmu.ac.th; 3 Department of Pharmacology, Faculty of Medicine, Chiang Mai University, Chiang Mai 50200, Thailand; seewaboon.s@cmu.ac.th; 4 Clinical Research Center for Food and Herbal Product Trials and Development (CR-FAH), Faculty of Medicine, Chiang Mai University, Chiang Mai 50200, Thailand

**Keywords:** type 2 diabetes, Thai herbal medicine, antihyperglycemic, anti-oxidant, remedy

## Abstract

This study aims to investigate selected medicinal plants’ anti-oxidative and antihyperglycemic activities to develop an effective remedy for lowering blood glucose levels and/or reducing diabetes complications. Thai medicinal plants, reported to have blood sugar-lowering effects, were selected for the study: *Coccinia grandis*, *Gymnema inodorum*, *Gynostemma pentaphyllum*, *Hibiscus sabdariffa*, *Momordica charantia*, *Morus alba*, and *Zingiber officinale*. Each species was extracted by Soxhlet’s extraction using ethanol as solvent. The ethanolic crude extract of each species was then evaluated for its phytochemicals, anti-oxidant, and antihyperglycemic activities. The results showed that the extract of *Z. officinale* gave the highest values of total phenolic and total flavonoid content (167.95 mg gallic acid equivalents (GAE)/g and 81.70 mg CE/g, respectively). Anti-oxidant activity was determined using DPPH and ABTS radical scavenging activity. Among the ethanolic extracts, *Z. officinale* exhibited the highest anti-oxidant activity with IC_50_ values of 19.16 and 8.53 µg/mL, respectively. The antihyperglycemic activity was assessed using α-glucosidase inhibitory and glucose consumption activities. *M. alba* and *G. pentaphyllum* demonstrated the highest α-glucosidase inhibitory activity among the ethanolic extracts, with IC_50_ values of 134.40 and 329.97 µg/mL, respectively. *Z. officinale* and *H. sabdariffa* showed the highest percentage of glucose consumption activity in induced insulin-resistant HepG2 cells at a concentration of 50 µg/mL with 145.16 and 107.03%, respectively. The results from α-glucosidase inhibitory and glucose consumption activities were developed as an effective antihyperglycemic remedy. Among the remedies tested, the R1 remedy exhibited the highest potential for reducing blood glucose levels, with an IC_50_ value of 122.10 µg/mL. Therefore, the R1 remedy should be further studied for its effects on animals.

## 1. Introduction

Diabetes mellitus is a chronic disease. The global diabetic population was 537 million (aged 20–79 years) in 2021 (10.5%); it has been estimated that this number will rise to 643 million (11.3%) in 2030 and 783 million (12.2%) in 2045 [1]. Type 2 diabetes mellitus (T2DM), affecting 95% of people, is caused by increased blood glucose levels due to insufficient pancreatic insulin secretion or insulin resistance in body cells [2]. Additionally, the oxidative stress caused by increased free radical production and decreased anti-oxidant defenses contributes to T2DM and increases the risk of complications related to diabetes mellitus [3]. The enzyme α-glucosidase hydrolyzes oligosaccharides or disaccharides into glucose molecules, essential for regulating postprandial blood glucose levels, and its inhibitors can decrease postprandial hyperglycemia [4]. Moreover, insulin is a hormone encouraging glucose uptake by acting on various target tissues, regulating blood glucose levels. Increasing glucose uptake to its target tissues can reduce hyperglycemia, which has been used to prevent or treat type 2 diabetes [5]. Antidiabetic drugs such as insulin, metformin, repaglinide and acarbose are available to reduce, control and manage diabetes mellitus by working on these mechanisms. However, some side effects may occur with the use of these drugs including hypoglycemia, nausea, diarrhea and weight gain [6]. In T2DM, oxidative stress resulting from increased production of free radicals and reduced antioxidant defenses can worsen the condition and increase the risk of complications [3]. Several factors including ineffective medication therapies, poor patient adherence to treatment and insufficient care regimens might increase the frequency of disease-related complications. Patients with type 2 diabetes may need to make considerable dietary and lifestyle modifications in addition to taking antidiabetic drugs. Therefore, antihyperglycemic complementary and alternative medicine, especially herbal remedies, may be alternative ways for patients with diabetes to reduce blood sugar levels and/or increase their quality of life. The phytochemicals found in medicinal plants such as alkaloids, phenolics, flavonoids, saponins and other compounds have been shown to be responsible for antihyperglycemic and anti-oxidant activity, especially phenolics and flavonoids, which are able to decrease the complications related to diabetes mellitus [7]. *C. grandis*, *G. inodorum*, *G. pentaphyllum*, *H. sabdariffa*, *M. charantia*, *M. alba*, and *Z. officinale* have the potential to decrease blood glucose levels. They accomplish this by increasing insulin secretion, enhancing glucose uptake, reducing α-glucosidase and exerting an anti-oxidant effect [8,9,10,11,12,13,14]. Several glucose-lowering mechanisms exist in type 2 diabetes, such as increased insulin secretion, glucose uptake, and α-glucosidase inhibition [6]. A combination of potential medicinal plants with different mechanisms will be beneficial for lowering glucose. Anti-oxidants may reduce the risk of diabetes complications by countering oxidative stress, in addition to their hypoglycemic effect. Therefore, this study aims to investigate the anti-oxidants and antihyperglycemic activities of the selected medicinal plants to develop an effective remedy for lowering blood glucose levels and/or reducing diabetes mellitus complications.

## 2. Results

### 2.1. Extraction Yield and Quality Control of Selected Medicinal Plants

The extraction yields of these medicinal plant extracts ranged from 12.05 to 29.52% in the following decreasing order: *G. inodorum* > *H. sabdariffa* > *M. alba* > *G. pentaphyllum* > *M. charantia* > *C. indica* > *Z. officinale* (Table 1). Among the ethanolic extracts, *Z. officinale* had the highest values of total phenolic content and total flavonoid content, with values of 167.95 ± 0.11 mg GAE/g extract and 81.70 ± 0.29 mg CE/g extract, respectively (Table 2).

### 2.2. Anti-Oxidant Activities of Crude Ethanolic Extracts

Anti-oxidant activity was assessed using DPPH and ABTS radical scavenging activities. The plant extracts showed inhibition in both antioxidant assays. *Z. officinale* revealed the highest anti-oxidant activity among the ethanolic extracts, with IC_50_ values of 19.16 ± 0.43 and 8.53 ± 0.04 µg/mL, respectively. As a positive control, ascorbic acid and trolox showed higher IC_50_ values than all extracts, with IC_50_ values of 3.79 ± 0.05 and 3.61 ± 0.01 µg/mL, respectively (Table 3).

### 2.3. Effect of Medicinal Plants on Cell Viability in Insulin-Resistant HepG2 Cells

**Figure 1:**
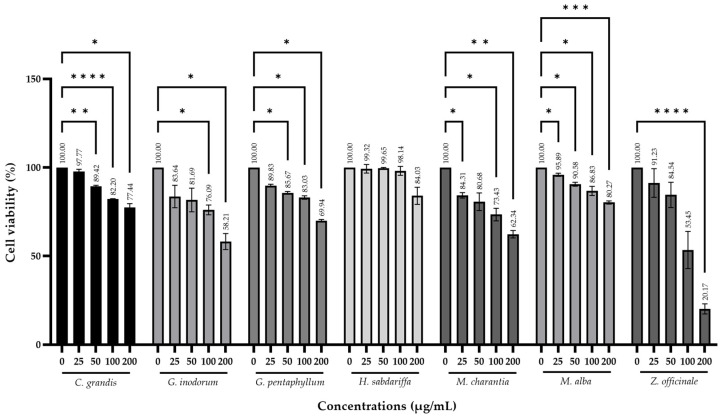
Effect of medicinal plants on cell viability in insulin-resistant HepG2 cells by SRB assay. The results were represented as mean ± SD (n = 3) and significantly different from the control group (untreated group) (* *p* < 0.05, ** *p* < 0.01, *** *p* < 0.001, **** *p* < 0.0001).

To determine the potential effects of medicinal plants on cell viability in a human hepatic cell line (HepG2), the cells were exposed to each extract ranging from 25 to 200 µg/mL for 24 h. After treatments, *Z. officinale* decreased cell viability with only 200 µg/mL showing a significant decrease. Additionally, *G. pentaphyllum*, *M. alba*, *G. inodorum*, *M. charantia* and *C. grandis* significantly decreased cell viability in a dose-dependent manner. On the other hand, *H. sabdariffa* did not induce any changes in cell viability compared with the control group (Figure 1). Furthermore, the concentrations of plant extract that did not exceed IC_20_ of cell viability value were used in further experiments: *Z. officinale*, *G. inodorum* and *M. charantia* at doses lower than 50 µg/mL; *C. grandis* and *G. pentaphyllum* at doses lower than 100 µg/mL and *H. sabdariffa* and *M. alba* at doses lower than 200 µg/mL, as shown in Table 4.

### 2.4. Anti-Hyperglycemic Activities of Crude Ethanolic Extracts

The anti-hyperglycemic activities were determined using α-glucosidase inhibitory and glucose consumption activities. *M. alba*, *G. pentaphyllum* and *C. grandis* showed the greatest potential α-glucosidase inhibitory activity among the ethanolic extracts with an IC_50_ value of 134.40 ± 0.26, 329.97 ± 3.70 and 423.73 ± 2.40 µg/mL, respectively, compared with the positive control, acarbose, which had an IC_50_ value of 571.27 ± 3.33 µg/mL. In contrast, Table 5 indicates that neither *M. charantia* nor *Z. officinale* had any inhibitory effect against the α-glucosidase enzyme.

**Figure 2:**
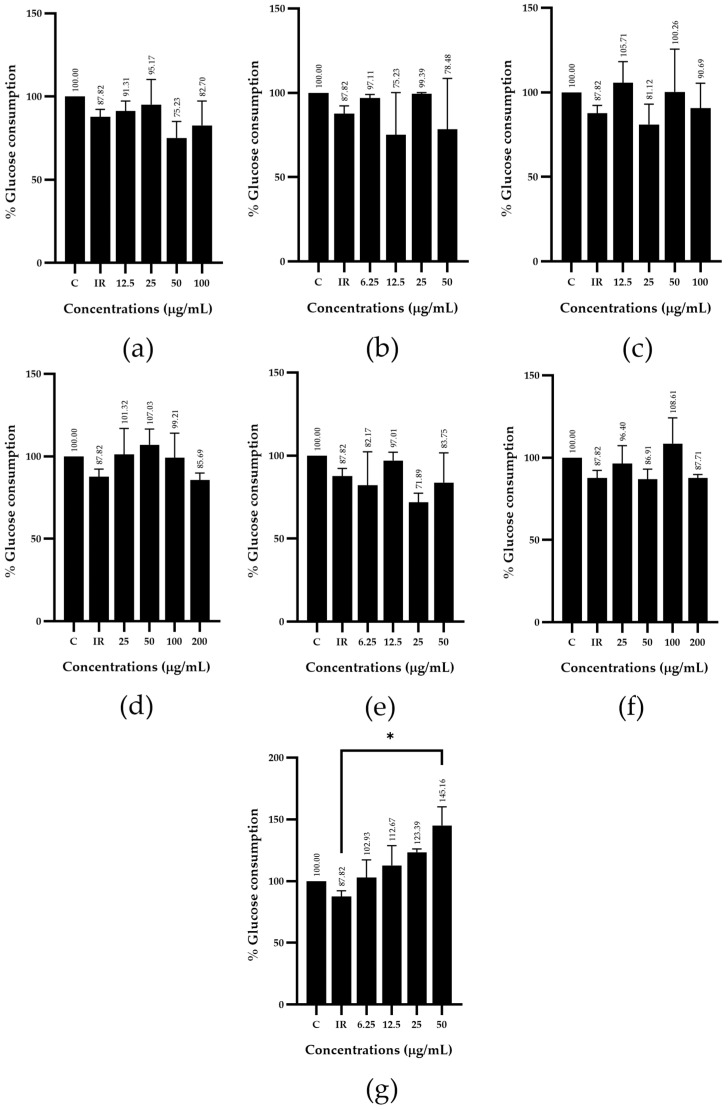
Glucose consumption percentage of ethanolic extracts; (**a**) *C. grandis* extract; (**b**) *G. inodorum* extract; (**c**) *G. pentaphyllum* extract; (**d**) *H. sabdariffa* extract; (**e**) *M. charantia* extract; (**f**) *M. alba* extract and (**g**) *Z. officinale* extract. The results were represented as mean ± SD (n = 3), and significantly differed from the control group (insulin-resistant group) (* *p* < 0.05).

In terms of the glucose consumption activity of these herbal medicines, *Z. officinale* (g) and *H. sabdariffa* (d) showed the highest potential percentage of glucose consumption activity via induced insulin-resistant HepG2 cells at a concentration of 50 µg/mL with 145.16 and 107.03%, respectively, when compared with the control group (insulin-resistant group), as shown in Figure 2.

### 2.5. Antihyperglycemic Activities of Remedies

The plant extracts were prepared as follows: R1 remedy included a 2:3 ratio of *G. pentaphyllum* to *M. alba*, R2 remedy included a 2:3 ratio of *H. sabdariffa* to *Z. officinale*, and R3 remedy included a 1:9 ratio of *M. alba* to *Z. officinale*. The antihyperglycemic activities were determined using α-glucosidase inhibitory and glucose consumption activities. The R1 remedy showed the greatest potential α-glucosidase inhibitory activity, with an IC_50_ value of 122.10 ± 0.17 µg/mL, when compared with the positive control (acarbose), which had an IC_50_ value of 571.27 ± 3.33 µg/mL. In contrast, the R3 remedy showed non-inhibition of the α-glucosidase enzyme, as shown in Table 6.

**Figure 3:**
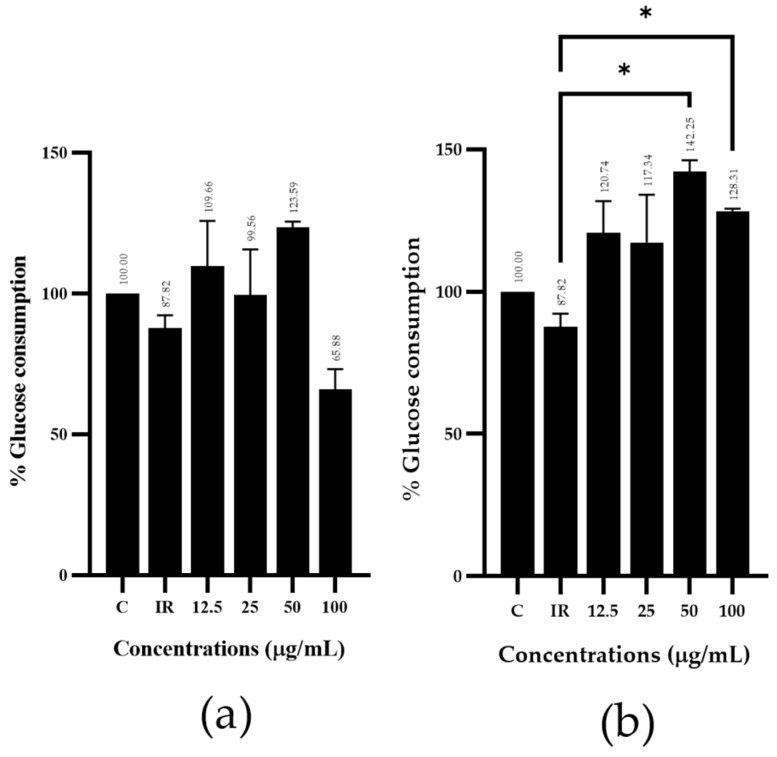
Glucose consumption percentage of remedies: (**a**) R2 remedy and (**b**) R3 remedy. The results were represented as mean ± SD (n = 3) and significantly differed from the control group (insulin-resistant group) (* *p* < 0.05).

Regarding the glucose consumption activity test via induced insulin-resistant HepG2 cells, *Z. officinale* extract showed the highest potential percentage of glucose consumption activity compared with that of the control group (insulin-resistant group) at doses of 6.25, 12.5, 25 and 50 µg/mL with 102.93, 112.67, 123.39 and 145.16%, respectively (Figure 2g). In contrast, the remedy including a mixture of plants was less effective than the single herbal extract. The test results indicated that the R3 remedy had a more pronounced effect on glucose consumption compared with that of the control group (insulin-resistant group). In addition, the R3 remedy (b), administered at test substance doses of 12.5, 25, 50 and 100 µg/mL, exhibited glucose consumption percentages of 120.74, 117.34, 142.25 and 128.31%, respectively. In contrast, the R2 remedy (a), at test substance doses of 12.5, 25, 50 and 100 µg/mL, resulted in glucose consumption percentages of 109.66, 99.56, 123.59 and 65.88%, respectively (Figure 3).

### 2.6. Physical-Chemical Properties of R1 Remedy

Physical-chemical analyses, including loss on drying, foreign matter, acid-insoluble ash, total ash, ethanol-soluble extractive, water-soluble extractive and total crude saponins content of the R1 remedy are presented in Table 7, along with their mean values.

### 2.7. Phytochemical Analysis by Thin-Layer Chromatography (TLC)

**Figure 4:**
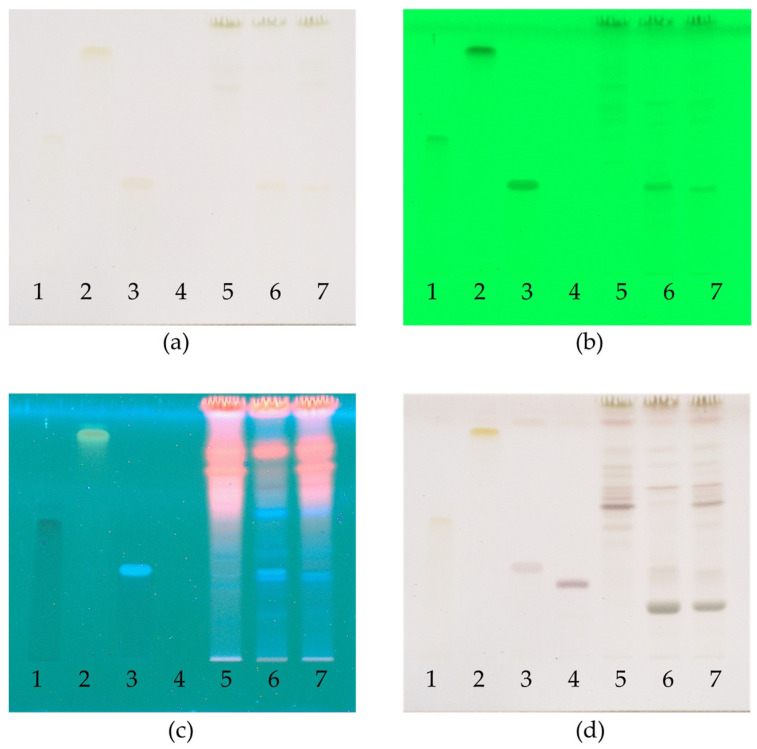
TLC chromatograms of ethanolic extract of remedy: (**a**) visible light; (**b**) UV at 254 nm; (**c**) UV at 366 nm; (**d**) 20% sulfuric acid spraying reagent; (1) rutin; (2) quercetin; (3) chlorogenic acid; (4) ginsenoside Rb1; (5) *G. pentaphyllum* extract; (6) *M. alba* extract and (7) the R1 remedy.

**Figure 5:**
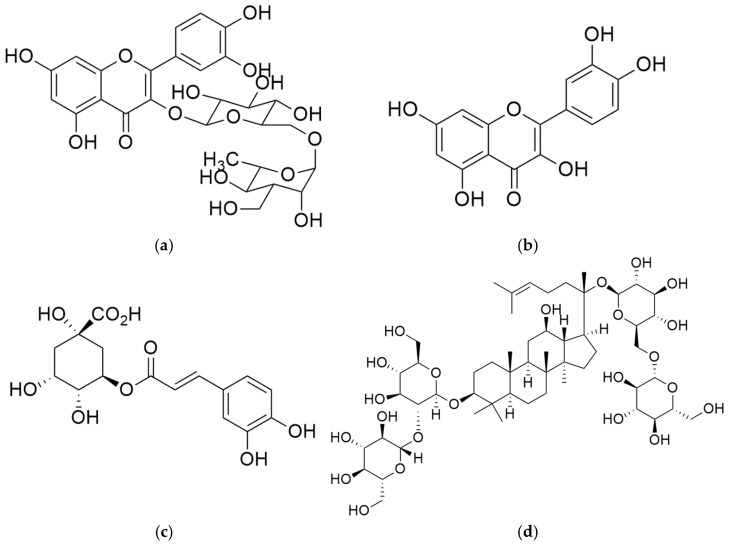
Compound structures of standard references: (**a**) rutin, (**b**) quercetin, (**c**) chlorogenic acid and (**d**) ginsenoside Rb1.

The chemical profiles of the ethanolic extracts of *G. pentaphyllum* and *M. alba*, along with the R1 remedy, were analyzed using the method outlined by THP 2021 [15]. This involved using TLC with a detector under UV light at wavelengths of 254 nm and 366 nm. After that, the plate was sprayed with 20% sulfuric acid in methanol, and heated at 105 °C for 10 min (Figure 4). Rutin, quercetin, chlorogenic acid, and ginsenoside Rb1 were used as the reference standards (Figure 5).

**Figure 6:**
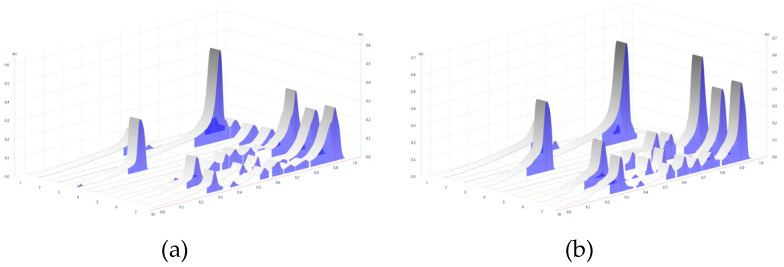
Densitograms of the remedy: (**a**) UV 254 nm, (**b**) UV at 366 nm, (1) rutin, (2) quercetin, (3) chlorogenic acid, (4) ginsenoside Rb1, (5) *G. pentaphyllum* extract, (6) *M. alba* extract and (7) the R1 remedy.

As a result, the remedy contained a mixture of chemical constituents derived from two different species and showed 11 spots on TLC. The major component (Rf 0.33) was derived from the *M. alba* leaf. Minor components were detected in varying amounts at Rf 0.19, 0.51, 0.55, 0.57, 0.64, 0.70, 0.72, 0.75, 0.78 and 0.88 in the compounds present in the *G. pentaphyllum* aerial part and the *M. alba* leaf. Compared with chemical standards, chlorogenic acid (Rf 0.33) was identified as the major component of the remedy. Rutin (Rf 0.51) was revealed as a minor component. Quercetin (Rf 0.84) and ginsenoside Rb1 (Rf 0.27) were undetected in the remedy based on the TLC pattern, as shown in Figure 4 and Figure 6. The ethanolic extracts of *M. alba* and the R1 remedy contained chlorogenic acid. *M. alba* and the R1 remedy had chlorogenic acid content values of 5.10 and 2.74 µg/mg extract, respectively.

## 3. Discussion

Diabetes mellitus, impacting patients’ daily lives and increasing their risk of developing other diseases, is one of the foremost global health crises of this decade [1]. T2DM is characterized by high blood glucose concentrations. This occurs due to insufficient pancreatic insulin secretion or insulin resistance in body cells. It can lead to severe complications in multiple organs and affects 95% of the general population [2]. Therefore, the main objective of T2DM treatment is to decrease blood glucose variations. Phytochemicals found in medicinal plants, such as phenolics, flavonoids, alkaloids, saponins and other compounds are responsible for antihyperglycemic and anti-oxidant activity, especially phenolics and flavonoids, which can decrease the complications related to diabetes mellitus [7,16]. Herbal remedies, among other complementary and alternative medicines, may offer patients with diabetes an alternative means to reduce blood sugar levels and improve their quality of life [17,18]. A combination of potential medicinal plants with different mechanisms will be beneficial for lowering glucose. Additionally, anti-oxidants may reduce the risk of diabetes complications by countering oxidative stress. Therefore, this study investigated the potential antihyperglycemic and anti-oxidant activity of selected medicinal plant extracts through various in vitro studies.

As a result, the yields of the medicinal plant extracts ranged from 12.05 to 29.52%. Among the ethanolic extracts, *G. inodorum* produced the highest yield. In the analysis of phytochemicals, *Z. officinale* extract demonstrated the highest levels of total phenolic and total flavonoid content among the ethanolic extracts with values of 167.95 ± 0.11 mg of GAE per gram of extract and 81.70 ± 0.29 mg of catechin equivalents (CE) per gram of extract, respectively. The anti-oxidant effects were assessed using DPPH and ABTS radical scavenging activities. DPPH and ABTS assays are commonly used analytical techniques for determining antioxidative activity in food, beverages, and plant extracts because of their rapidness, sensitivity, and reproducibility [19,20,21,22]. The results indicate that *Z. officinale* exhibited the highest anti-oxidant activity among the ethanolic extracts, with IC_50_ values of 19.16 ± 0.43 and 8.53 ± 0.04 µg/mL, respectively. This was in comparison to the standard anti-oxidants, ascorbic acid, and trolox, which had IC_50_ values of 3.79 ± 0.05 and 3.61 ± 0.01 µg/mL, respectively. These findings align with previous reports by Stoilova et al. [23] and Gaurav et al. [24], indicating that plant extracts with high phenolic and flavonoid content exhibit stronger anti-oxidant activity. Mošovská et al. [25] and Amir et al. [26] suggested that ginger is a rich source of polyphenols, containing high levels of total phenolics and flavonoids with potent anti-oxidant properties. The effect of anti-oxidants on DPPH and ABTS is reportedly due to their hydrogen-donating ability [27,28,29]. The study showed that the extracts can donate protons and act as free radical inhibitors or scavengers. This means they could potentially function as antioxidant agents, even though their free radical scavenging activities were lower than those of the standard. Anti-oxidants may lower the risk of complications among patients with diabetes mellitus by defending against oxidative stress. Therefore, plant extracts with high anti-oxidant activities were selected.

Plant extracts containing phytochemicals have been associated with potential beneficial and toxic effects. Cell-based studies are commonly used to detect cytotoxic and other adverse effects with highly cost-effective screenings [30]. The liver is one of the primary insulin target organs and is the main source of endogenous glucose production [31]. Several studies have used hepatoma cell lines, especially HepG2 cells, to study hepatocytes in insulin signaling or metabolism [32]. The study found that after treatment with plant extracts at concentrations ranging from 25 to 200 µg/mL, *Z. officinale* significantly reduced cell viability at a concentration of 200 µg/mL. This is consistent with the findings of Ruangnoo et al. [33]. Additionally, *G. pentaphyllum*, *M. alba*, *G. inodorum*, *M. charantia* and *C. grandis* significantly decreased cell viability in a dose-dependent manner. However, the extract of *H. sabdariffa* did not induce changes in cell viability, consistent with a related study [34]. For safety, the concentrations used in the glucose consumption test of each plant extract did not exceed the IC_20_ of cell viability.

The antihyperglycemic effect was determined using α-glucosidase inhibitory and glucose consumption activities. The enzyme α-glucosidase is primarily responsible for digesting carbohydrates; it hydrolyzes the linkages of oligosaccharides or disaccharides and releases glucose molecules [35]. Thus, its inhibitors can reduce postprandial hyperglycemia and have been used to prevent or treat T2DM [36,37]. The results indicate that *M. alba* and *G. pentaphyllum* exhibited the potential α-glucosidase inhibitory activity among the ethanolic extracts, with IC_50_ values of 134.40 ± 0.26 and 329.97 ± 3.70 µg/mL, respectively. In comparison, the acarbose group showed IC_50_ values of 571.27 ± 3.33 µg/mL. These findings align with a related report by Kim et al. [38]. Additionally, Hwang et al. [39] reported that mulberry leaves in Chinese herbal remedies are potent α-glucosidase inhibitors, with a level four times higher than that of acarbose. Megalli et al. [40] reported that five-leaf ginseng extract can inhibit α-glucosidase with an IC_50_ of 53.9 µg/mL. According to the present study, *M. alba* and *G. pentaphyllum* extracts can inhibit α-glucosidase to a higher degree than acarbose, proving to be potential plants for decreasing blood glucose level.

The results of glucose consumption using HepG2 cells in this study indicated that ethanolic extract of *Z. officinale* and *H. sabdariffa* at a concentration of 50 µg/mL increased glucose consumption in HepG2 cells with 145.16 and 107.03%, respectively, compared with the control group (insulin-resistant group). This suggests that both extracts have potential by increasing glucose consumption. Similarly, the related study by Li et al. [41] reported significantly increased glucose consumption in HepG2 cells by 20 to 30% using ginger extract at concentrations of 50 and 75 µg/mL, compared with the control group.

Patients with type 2 diabetes may require antidiabetic medications, along with significant dietary and lifestyle adjustments. Ineffective medication regimens, patient non-adherence and insufficient care may lead to more disease-related complications [3]. Herbal remedies are increasingly considered by people with diabetes to improve their quality of life and reduce their blood sugar levels. To achieve this objective, the development of antihyperglycemic remedies focuses on using herbal medicine with various mechanisms to lower glucose levels, such as increasing insulin secretion, enhancing glucose uptake and inhibiting α-glucosidase. In this study, *M. alba* and *G. pentaphyllum* exhibited potential α-glucosidase inhibitory activity. *Z. officinale* and. *H. sabdariffa* exhibit potential by increasing glucose consumption. Therefore, these plant extracts were prepared as follows: the R1 remedy included *G. pentaphyllum* and *M. alba* to enhance the α-glucosidase inhibitory effect, the R2 remedy included *H. sabdariffa* and *Z. officinale* to enhance the glucose consumption effect, and the R3 remedy included *M. alba* and *Z. officinale* to enhance the α-glucosidase inhibitory and glucose consumption effects. The results indicate that the R1 remedy exhibited potential α-glucosidase inhibitory activity greater than a single herbal extract. Furthermore, the R3 remedy shows potential by increasing glucose consumption without any significant effect with *Z. officinale* extract. Nevertheless, the beneficial effect of mulberry leaf extract in inhibiting the oxidation process is related to an increased risk of diabetes complications due to hyperglycemia. Moreover, the results indicated that the R3 remedy significantly affected glucose consumption, more than the R2 remedy. Therefore, the R3 remedy is worthy of note and should be further studied. In conclusion, the effect of anti-oxidants, α-glucosidase inhibitory action and glucose consumption of the plant should be considering in the selection of remedies. Accordingly, the R1 remedy demonstrated the potential to effectively lower blood glucose levels, potentially reducing the risk of disease complications due to oxidative stress in T2DM.

Certain herbal medicines have been found to contain irregularities in their raw materials, directly impacting their effectiveness and safety. To ensure quality control, authenticating herbal drug substances and verifying their authenticity is crucial before further processing. Pharmacopeial monographs use organoleptic, macroscopic and microscopic analyses, as well as chemical assessments of raw plant materials, to determine the botanical origins of crude drugs [42,43]. The crude drugs in the remedies, R1, R2, and R3, were easily identified based on plant morphology. The plant species used in this study are well-known herbs, but only *C. grandis* was not officially established in the Thai Herbal Pharmacopoeia (THP) 2021 [15]. According to a physico-chemical examination, i.e., loss on drying, foreign matter, acid-insoluble ash, total ash, ethanol-soluble extractive value, water-soluble extractive value and total crude saponins content of the crude drugs used in the remedies, were used for quality evaluations. As a result, the quality of each species was accepted according to the THP 2021. Physico-chemical examination of the potential R1 remedy showed the average constant value of the combination of two species: *G. pentaphyllum* and *M. alba* (Table 7). These data will be a part of the criteria to determine the quality of this remedy for further study.

TLC is commonly used for characterizing plant extracts, essential oils and other plant-derived products [44]. The chemical profiles of the ethanolic extract of *G. pentaphyllum*, *M. alba* and the remedy compounded from them were evaluated using the TLC and detection under visible light, UV light at 254 and 366 nm and 20% sulfuric acid in methanol spraying reagent (Figure 4). The chemical constituents found in the remedy included a mixture of these two species. The major component (Rf 0.33) originated from the *M. alba* leaf. The minor components were found at Rf 0.19, 0.51, 0.55, 0.57, 0.64, 0.70, 0.72, 0.75, 0.78 and 0.88. Compared with chemical standards, the remedy found chlorogenic acid (Rf 0.33) as a major component, and rutin (Rf 0.51) was revealed as a minor component (Figure 4 and Figure 6). The chemical constituents reported in *G. pentaphyllum* and *M. alba* were gypenoside, *1*-deoxynojirimycin, ellagic acid, protocatechuic acid, gallic acid, chlorogenic acid, vanillic acid, ferulic acid, rutin, quercetin, kaempferol and catechin [12,45,46,47,48,49,50]. The present study used rutin, quercetin, chlorogenic acid, and ginsenoside Rb1 as standard references. Quercetin and ginsenoside Rb1 were undetected in the TLC chromatogram, possibly due to their lower concentration in the remedy. The R1 remedy contains crude saponin content (2.33 ± 0.06%) from *G. pentaphyllum* although ginsenoside Rb1 cannot be found in TLC. Related studies have shown that it can reduce postprandial glucose levels in obese Zucker fatty rats by inhibiting α-glucosidase activity [40]. Phytochemicals found in *G. pentaphyllum* included approximately 90 dammarane-type glycosides, contributing to its pharmacologic activities. Moreover, ginsenoside Rg1 reveals inhibition of α-glucosidase activity [51]. Ginsenoside Rb1 can increase the translocation of glucose transporters (GLUTs) to increase glucose uptake in adipocytes [52]. Ginsenoside Rb1 and quercetin found in *G. pentaphyllum* can inhibit α-glucosidase with 55.3 and 75.7% at 40 and 10 µg/mL doses, respectively [53]. Ginsenoside Rb2 can inhibit α-glucosidase with an IC_50_ value of 32.2 µM and significantly increase glucose consumption in HepG2 cells [54]. Furthermore, chlorogenic acid and rutin can decrease plasma glucose levels after treatment in diabetic rats for 11 days, as much as one half of the observed antidiabetic activity of mulberry leaf [55]. Chlorogenic acid, vanillic acid, ferulic acid and rutin led to potent α-glucosidase inhibitory activities even when the concentration was five-fold diluted; they still revealed higher inhibitory activity than *Pyrus ussuriensis* extract [56]. Rutin and quercetin compounds isolated from mulberry fruit have potential antidiabetic effects via increased glucose uptake in 3T3-L1 adipocytes [57]. Therefore, chlorogenic acid and rutin were used as biomarkers in this study. Chromatographic techniques can provide valuable chemical profiles for quality control and for confirming the authenticity of herbal products and raw materials [42]. The remedy specifications presented in this study can be used as guidelines for quality control and reference in further research.

## 4. Materials and Methods

### 4.1. Plant Materials

The plant materials were selected using the criteria listed below.

(1) Revealed potent antihyperglycemic potential, especially in clinical trials or in-vivo study.
(2) Indigenous plants or easily found in Thailand.
(3) Nontoxic.

They were collected in Thailand, especially Chiangmai Province. Each plant was authenticated by an expert botanist and compared with the voucher specimen deposited in the herbarium at the Faculty of Pharmacy, Chiang Mai University, Thailand (Table 8).

### 4.2. Preparation of Plant Extracts

The ethanolic extract of these plants was prepared following the method described in a previous study [58]. The plant materials were dried in a hot air oven at 50 °C until the moisture was less than 10% *w*/*w*. The dried materials were ground into a coarse powder using a grinder. They were then extracted with 95% ethanol at a ratio of 1:10 (*w*/*v*) for 24 h in a Soxhlet’s apparatus (temperature approximately 80 °C; boiling point of ethanol 78.37 °C) and evaporated using a rotary evaporator (BUCHI, R-300, Büchi Labortechnik AG, Flawil, Switzerland) at 40 to 50 °C under reduced pressure. The crude extracts were stored in an airtight container at 4 °C until used in further experiments. The weight of crude extracts was calculated as yield percentage using Equation (1) as shown below.

% Yield = [(A_0_/A_1_] × 100 (1)

where A_0_ and A_1_ are weight in grams of plant extract and dried powder, respectively.

It is determined by dividing the weight of the dried extract by the weight of the dried plant sample and multiplying it by 100. This formula provides a direct calculation of the dried extract that was obtained.

### 4.3. Quality Control of Phytochemicals

#### 4.3.1. Determination of Total Phenolic Content

The Folin-Ciocalteu method was modified to determine the total phenolic content [59]. Briefly, 100 µL of 10% Folin-Ciocalteu reagent and 80 µL of 7.5% Na_2_CO_3_ were combined with 20 µL of various concentration samples, then the mixture was incubated for 30 min at room temperature. The absorbance was measured using a microplate reader (BioTek, Winooski, VT, USA) at 765 nm. Gallic acid was used as the standard. The results were presented as mg gallic acid equivalents in g of extract (mg GAE/g extract).

#### 4.3.2. Determination of Total Flavonoid Content

The aluminum chloride colorimetric method with some modifications was used to calculate the total flavonoid content [60]. Briefly, 7.5 µL of 5% NaNO_2_ was added to 25 µL of various concentration samples and stood at room temperature for 6 min. Then, 15 µL of 10% AlCl_3_ was added and stood at room temperature for 6 min. Then, 152.5 µL of distilled water was added together with 50 µL of 1 M NaOH. The absorbance was measured using a microplate reader (BioTek, Winooski, VT, USA) at 532 nm, using catechin as the standard. The results were presented as mg catechin equivalents in g of extract (mg CE/g extract).

### 4.4. Determination of Anti-Oxidant Activities

#### 4.4.1. DPPH (2,2-Diphenyl-1-Picrylhydrazyl) Radical Scavenging Assay

The colorimetric method with some modifications was used to evaluate DPPH radical scavenging activity [61]. Briefly, 180 µL of 0.2 mM DPPH in methanol was added to 20 µL of various concentration samples, and the mixture was then incubated for 30 min at room temperature in the dark. The absorbance was measured using a microplate reader (BioTek, Winooski, VT, USA) at 517 nm, using ascorbic acid as the standard. The anti-oxidant activity of the sample was represented as the IC_50_, and the inhibition percentage was calculated using Equation (2) below.

% Inhibition = [(A_0_ − A_1_)/A_0_] × 100 (2)

where A_0_ and A_1_ are absorbances of the control and sample, respectively.

#### 4.4.2. ABTS (2,2-Azinobis-(3-Ethylbenzothiazoline-6-Sulphonate)) Radical Scavenging Assay

The colorimetric method with some modifications was used to determine the ABTS radical scavenging activity [61]. A total of 7.4 mM of ABTS^•+^ stock solution was mixed with 2.45 mM of K_2_S_2_O_8_ at a ratio of 1:1 *v*/*v* after which the reaction was incubated for 12 h at room temperature in the dark. Distilled water was used to dilute the ABTS^•+^ reagent to an absorbance of 0.700 ± 0.02 at 734 nm. Briefly, 180 µL of ABTS^•+^ reagent was combined with 20 µL of various concentration samples, and the mixture was incubated for 6 min in the dark. The absorbance was measured using a microplate reader (BioTek, Winooski, VT, USA) at 734 nm, using trolox as the standard. The anti-oxidant activity of the sample was represented as the IC_50_, and the inhibition percentage was calculated using Equation (3) below.

% Inhibition = [(A_0_ − A_1_)/A_0_] × 100 (3)

where A_0_ and A_1_ are absorbances of the control and sample, respectively.

### 4.5. Determination of Anti-Hyperglycemic Activities

#### 4.5.1. The α-Glucosidase Inhibitory Assay

The colorimetric method with some modifications was used to calculate the α-glucosidase inhibitory activity [62]. Briefly, a mixture of 10 μL of sample in DMSO, 40 μL of 0.1 M phosphate buffer (pH 6.9), and 100 μL of 0.1 U α-glucosidase was incubated at 37 °C for 10 min. The mixture was then added to 50 μL of 0.1 mM *p*-nitrophenyl-α-D-glucopyranoside followed by an additional 20 min incubation at 37 °C. The absorbance was measured using a microplate reader (BioTek, Winooski, VT, USA) at 405 nm, using acarbose as the standard. The α-glucosidase inhibitory activity was represented as the IC_50_, and the inhibition percentage was calculated using Equation (4) below.

% Inhibition = [(A_0_ − A_1_)/A_0_] × 100 (4)

where A_0_ and A_1_ are absorbances of the control and sample, respectively.

#### 4.5.2. Cell Culture and Insulin-Resistant HepG2 Cell Model (IRM) Cell Culture

Human HepG2 cells were purchased from the American Type Culture Collection (ATCC, Manassas, VA, USA) and cultured in Dulbecco’s modified Eagle’s medium (DMEM) medium supplemented with 10% fetal bovine serum (FBS) and 1% penicillin-streptomycin; the cells were stored at 37 °C with 5% CO_2_ in a humidified environment. After 24 h of cell culture in 96-well plates, the medium was changed to a non-FBS medium containing 5 × 10^−7^ mol/L insulin for 24 h, and the cells were used in further experiments [63].

#### 4.5.3. Noncytotoxic Evaluation

The SRB colorimetric assay with modifications was used to calculate the non-cytotoxic evaluation [64]. HepG2 cells were cultured on 96-well plates at 5 × 10^3^ cells/well overnight. The cells were incubated with various concentrations of sample for 24 h. Then, proteins were precipitated using a final concentration of 10% of trichloroacetic acid overnight. The precipitated proteins were washed and stained with 100 μL of SRB solution for 1 h, after which the excess dye was removed three times and repeatedly washed with 1% (*v*/*v*) of acetic acid. The protein-bound dye was dissolved in 100 mM Tris-based solution. The absorbance was then measured using a microplate reader (BioTek, Winooski, VT, USA) at 510 nm. The viability percentage was calculated using Equation (5) shown below.

% Viability = [A_1_/A_0_] × 100 (5)

where A_0_ and A_1_ are absorbances of the control and sample, respectively.

#### 4.5.4. Glucose Consumption Assay

The enzymatic method was used to determine the glucose uptake activity [63]. After the incubation period, the medium was collected to determine glucose consumption using a glucose assay kit (MAK263-1KT; Sigma-Aldrich, Darmstadt, Germany)) according to the manufacturer’s protocol. The glucose consumption percentage was calculated using Equation (6) shown below.

% Glucose consumption = [A_0_ − A_1_/A_0_] × 100 (6)

where A_0_ and A_1_ are absorbances of blank and sample, respectively.

### 4.6. Physical-Chemical Properties of Remedy

The physical and chemical properties of the medication were assessed using the official procedures outlined in the THP 2021 [15].

#### 4.6.1. Determination of Loss on Drying

Three grams of dried plant powder was weighed into previously weighed weighing bottles and dried at 105 °C until the weight was constant. After that, the weighing bottles were left to cool in a desiccator, then weighed and the weight loss was calculated and shown in percentage.

#### 4.6.2. Determination of Foreign Matter

One hundred grams of plant material was spread thinly on a tray. The foreign matter was separated by visual inspection as far as possible and then weighed. The content of foreign matter was calculated and shown in percentage.

#### 4.6.3. Determination of Total Ash

Three grams of dried plant powder was weighed in previously weighed crucibles and incinerated at 450 °C in an electrical muffle furnace (Thermo Fisher Scientific, Waltham, MA, USA) until it became white. After that, the crucible was dried at 105 °C, left to cool in a desiccator and weighed without delay. The content of total ash was calculated and shown in percentage.

#### 4.6.4. Determination of Acid-Insoluble Ash

Twenty-five milliliters of hydrochloric acid (2 M) was added to the crucibles, which contained ash from the total ash, then covered with a watch glass and boiled for 5 min on a water bath. The insoluble matter was collected on Whatman filter paper No. 40 and washed with hot water until the filtrate was neutral. After that, they were transferred to the original crucible, dried on a hot plate, and then incinerated at 500 °C in an electrical muffle furnace (Thermo Fisher, Waltham, MA, USA). The crucibles were dried at 105 °C, left to cool in a desiccator and weighed without delay. The content of acid-insoluble ash was calculated and shown in percentage.

#### 4.6.5. Determination of Ethanol-Soluble Extractive Value

Five grams of dried plant powder was macerated with 100 mL of 95% ethanol in a closed conical flask. After that, the conical flask was placed in a shaker for 6 h and stood for 18 h. The marc was rapidly filtered using Whatman filter paper No. 1. After that, 20 mL of the filtrate was transferred to pre-weighed evaporating dishes and evaporated to dryness using a water bath. The extract was dried at 105 °C, left to cool in a desiccator and then weighed without delay. The content of extractable matter was calculated and shown as a percentage.

#### 4.6.6. Determination of Water-Soluble Extractive Value

Five grams of dried plant powder was macerated with 100 mL of chloroform water in a closed conical flask. After that, the conical flask was placed in a shaker for 6 h and stood for 18 h. The marc was rapidly filtered using Whatman filter paper No. 1. After that, 20 mL of the filtrate was transferred to pre-weighed evaporating dishes and evaporated to dryness on a water bath. The extract was dried at 105 °C, left to cool in a desiccator and then weighed without delay. The content of extractable matter was calculated and shown in percentage.

#### 4.6.7. Determination of Total Crude Saponins

Five hundred milligrams of dried plant powder was refluxed with 50 mL of water in a round-bottomed flask for 2 h. The marc was filtered and washed with 40 mL of hot water using Whatman filter paper No. 1. The filtrate was adjusted to 100 mL with water, then 20 mL of the filtrate was transferred to a separator and extracted with 30 mL of 1-butanol. The 1-butanol extract was washed with 20 mL of water and discarded after the washing. After that, the 1-butanol extract was transferred to pre-weighed evaporating dishes and evaporated to dryness. The extract was dried at 105 °C, left to cool in a desiccator and then weighed without delay. The content of total crude saponin matter was calculated and shown in percentage.

#### 4.6.8. Phytochemical Analysis by TLC

##### Sample and Standard Preparation

The ethanolic extract of *G. pentaphyllum*, *M. alba*, and the R1 remedy (10 mg) was mixed with 1 mL of ethanol and then filtered. Solutions of standards, rutin, quercetin, chlorogenic acid, and ginsenoside Rb1 were prepared by dissolving 1 mg of each standard in 1 mL of ethanol to obtain a 1 mg/mL concentration.

##### Chemical Fingerprint

For the TLC fingerprint, a 3 µL ethanolic extract of sample and 3 µL of reference standard solution, rutin, quercetin, chlorogenic acid, and ginsenoside Rb1, were applied to the TLC plate by a TLC semiautomatic sampler Linomat 5 (CAMAG, Muttenz, Switzerland). The plate was then developed to a distance of 12 mm in a 12 × 10 cm glass twin trough chamber (CAMAG, Muttenz, Switzerland). The optimum mobile phase used was a mixture of chloroform/ethyl acetate/methanol/water in the ratio (15:40:22:9). The developed plate was air-dried and detected under visible light, UV light (254 and 366 nm) and chemical reagent (20% sulfuric acid in methanol), followed by heating for 10 min. Data from the finished chromatogram indicated the migrating behavior of the separated substances evaluated (7) as an Rf value.

Rf value = [a/b](7)

where a and b are the distance traveled by the component and distance traveled by the solvent, respectively.

##### Analysis of Chlorogenic Acid by TLC

Chromatography was conducted using 20 × 10 cm preactivated TLC Silica gel 60 F_254_ plates (Merck, Darmstadt, Germany). The ethanolic extract of the sample (conc. 10 mg/mL) and reference standard solution, chlorogenic acid (conc. 0.2–2 mg/mL), were applied to the TLC plate using a semiautomatic sampler Linomat 5 (CAMAG, Muttenz, Switzerland). The mobile phase consisted of chloroform/ethyl acetate/methanol/water (15:40:22:9). Linear ascending development was carried out in a twin glass chamber saturated with the mobile phase. Densitometric scanning was performed using a CAMAG scanner III at 254, 280, 320, and 366 nm.

### 4.7. Statistical Analysis

The results were represented as triplicate mean ± standard deviation (SD). All statistical analyses were performed using GraphPad Prism 9.0 Software (GraphPad Software, Inc., San Diego, CA, USA) using analysis of variance (ANOVA) with Tukey’s multiple comparison tests. The results were considered statistically significant at * *p* < 0.05, ** *p* < 0.01, *** *p* < 0.001 and **** *p* < 0.0001. The inhibitory concentration at 20% (IC_20_) and 50% (IC_50_) were performed using GraphPad Prism 9.0 software. At first, the concentration was turned to Log X, and then analyzed by the Nonlinear regression (curve fit) mode followed by selected Dose-response-inhibition and chose log (inhibitor) vs. normalized response–Variable slope.

## 5. Conclusions

The selected Thai medicinal plants, *C. grandis*, *G. inodorum*, *G. pentaphyllum*, *H. sabdariffa*, *M. charantia*, *M. alba*, and *Z. officinale* were investigated for their antioxidative and antihyperglycemic activities. The extract of *Z. officinale* had the highest total flavonoid content (81.70 mg CE/g) and total phenolic content (167.95 mg GAE/g) compared to other extracts. *Z. officinale* also exhibited the highest antioxidant activity with IC_50_ values of 19.16 and 8.53 µg/mL. Among the ethanolic extracts, *M. alba* and *G. pentaphyllum* showed the highest α-glucosidase inhibitory activity, with IC_50_ values of 134.40 and 329.97 µg/mL, respectively. Additionally, *Z. officinale* and *H. sabdariffa* demonstrated the highest percentage of glucose-consuming activity at 50 µg/mL, with 145.16% and 107.03%, respectively, in induced insulin-resistant HepG2 cells. The results from glucose consumption and α-glucosidase inhibitory effects were utilized to develop an effective antihyperglycemic remedy. The results showed that combining *G. pentaphyllum* and *M. alba* (R1 remedy) can improve the suppression of postprandial hyperglycemia. These findings may provide further evidence of the remedy’s potential antihyperglycemic properties. Consequently, the R1 remedy should be further investigated in vivo.

## Figures and Tables

**Table 1:** Extraction yield of ethanolic extracts.

Sample	Yield (% *w*/*w*)
*C. grandis*	13.68
*G. inodorum*	29.52
*G. pentaphyllum*	15.75
*H. sabdariffa*	28.32
*M. charantia*	15.11
*M. alba*	25.66
*Z. officinale*	12.05

**Table 2:** Total phenolic content, and total flavonoid content of ethanolic extracts.

Sample	Total Phenolic Content (mg GAE/g Extract)	Total Flavonoid Content (mg CE/g Extract)
*C. grandis*	57.60 ± 0.45	21.83 ± 0.35
*G. inodorum*	41.33 ± 0.65	11.92 ± 0.15
*G. pentaphyllum*	20.87 ± 0.07	5.07 ± 0.54
*H. sabdariffa*	48.75 ± 0.73	24.63 ± 0.16
*M. charantia*	29.00 ± 0.50	6.88 ± 0.49
*M. alba*	54.94 ± 0.51	35.41 ± 0.41
*Z. officinale*	167.95 ± 0.11	81.70 ± 0.29

GAE = gallic acid equivalents. CE = catechin equivalents. The results were represented as mean ± SD (n = 3).

**Table 3:** DPPH and ABTS radical scavenging activity of ethanolic extracts.

Sample	DPPH (IC_50_ (µg/mL))	ABTS (IC_50_ (µg/mL))
*C. grandis*	122.53 ± 0.21 ****	62.93 ± 1.02 ****
*G. inodorum*	317.80 ± 1.30 ****	93.00 ± 1.27 ****
*G. pentaphyllum*	694.37 ± 1.72 ****	554.00 ± 0.70 ****
*H. sabdariffa*	101.30 ± 0.62 ****	52.84 ± 0.42 ****
*M. charantia*	680.73 ± 1.37 ****	165.43 ± 0.46 ****
*M. alba*	89.13 ± 0.18 ****	56.03 ± 0.41 ****
*Z. officinale*	19.16 ± 0.43 ****	8.53 ± 0.04 ****
Ascorbic acid	3.79 ± 0.05	-
Trolox	-	3.61 ± 0.01

IC_50_ = Inhibitory concentration at 50%. The results were represented as mean ± SD (n = 3) and significantly different from the ascorbic acid (DPPH) and trolox (ABTS) (**** *p* < 0.0001).

**Table 4:** IC_20_ of cell viability in insulin-resistant HepG2 cells after treatment with medicinal plants.

Sample	IC_20_ (µg/mL)
*C. grandis*	152.10 ± 14.25
*G. inodorum*	54.63 ± 29.85
*G. pentaphyllum*	99.73 ± 0.22
*H. sabdariffa*	251.57 ± 86.98
*M. charantia*	49.71 ± 15.59
*M. alba*	200.77 ± 23.64
*Z. officinale*	55.68 ± 18.79

IC_20_ = Inhibitory concentration at 20%. The results were represented as mean ± SD (n = 3).

**Table 5:** α-glucosidase inhibitory activity of ethanolic extracts.

Sample	*α*-Glucosidase Inhibitory (IC_50_ (µg/mL))
*C. grandis*	423.73 ± 2.40 ****
*G. inodorum*	519.23 ± 4.58 ****
*G. pentaphyllum*	329.97 ± 3.70 ****
*H. sabdariffa*	>1000 ****
*M. charantia*	NI
*M. alba*	134.40 ± 0.26 ****
*Z. officinale*	NI
Acarbose	571.27 ± 3.33

IC_50_ = Inhibitory concentration at 50%. NI = No inhibition. The results were represented as mean ± SD (n = 3). Significantly different from the acarbose (**** *p* < 0.0001).

**Table 6:** α-glucosidase inhibitory activity of studied remedies.

Sample	*α*-Glucosidase Inhibitory (IC_50_ (µg/mL))
R1 remedy	122.10 ± 0.17 ****
R3 remedy	NI
Acarbose	571.27 ± 3.33

IC_50_ = Inhibitory concentration at 50%. NI = No inhibition. The results were represented as mean ± SD (n = 3), and significantly differed from the acarbose (**** *p* < 0.0001).

**Table 7:** Physical-chemical properties of the R1 remedy.

Specification	Content (% *w*/*w*)
Loss on drying	7.05 ± 0.02
Foreign matter	Not found
Acid-insoluble ash	1.41 ± 0.07
Total ash	10.64 ± 0.05
Ethanol-soluble extractive value	13.59 ± 0.08
Water-soluble extractive value	25.11 ± 0.01
Total crude saponins	2.33 ± 0.06

The results were represented as mean ± SD (n = 3).

**Table 8:** Medicinal plants used in this study.

No.	Scientific Name	Family	Part Used
1	*Coccinia grandis* (L.) Voigt	Cucurbitaceae	Aerial part
2	*Gymnema inodorum* (Lour.) Decne.	Asclepiadaceae	Leaf
3	*Gynostemma pentaphyllum* (Thunb.) Makino	Cucurbitaceae	Aerial part
4	*Hibiscus sabdariffa* L.	Malvaceae	Calyx
5	*Momordica charantia* L.	Cucurbitaceae	Fruit
6	*Morus alba* L.	Moraceae	Leaf
7	*Zingiber officinale* Roscoe	Zingiberaceae	Rhizome

## Data Availability

The raw data supporting the conclusions of this article will be made available by the authors on request.
